# Bovine Genome-Microbiome Interactions: Metagenomic Frontier for the Selection of Efficient Productivity in Cattle Systems

**DOI:** 10.1128/mSystems.00103-19

**Published:** 2019-05-07

**Authors:** Phillip R. Myer

**Affiliations:** a Department of Animal Science, University of Tennessee, Knoxville, Tennessee, USA

**Keywords:** cattle, metagenomics, microbiome, rumen

## Abstract

The mutualistic, commensal, and parasitic microorganisms that reside in the rumen and lower gastrointestinal tract of cattle and other ruminants exert enormous influence over animal physiology and performance. Because these microbial communities are critical for host nutrient utilization and contribute to the metabolic capacity of the rumen, past research has aimed to define host-microbe symbioses in cattle by examining the rumen and lower gut microbiomes with respect to production phenotypes, such as feed efficiency. However, as the field of bovine gut microbial ecology progresses, multidisciplinary approaches must be employed, combining host genomics and other omics-based techniques to understand the complex host-microbe network. In this perspective, I discuss the direction of the field of bovine gut microbial ecology with regard to feed efficiency and explore how the grand challenge of such research will be to maintain host-efficient gut microbiomes in cattle production through manipulations of genome-microbiome interactions.

## Abstract (precis)

The mutualistic, commensal, and parasitic microorganisms that reside in the rumen and lower gastrointestinal tract of cattle and other ruminants exert enormous influence over animal physiology and performance. Because these microbial communities are critical for host nutrient utilization and contribute to the metabolic capacity of the rumen, past research has aimed to define host-microbe symbioses in cattle by examining the rumen and lower gut microbiomes with respect to production phenotypes, such as feed efficiency.

## PERSPECTIVE

To address food security challenges, we must continue to actively seek and develop novel tools and resources to produce animal proteins in an economically, environmentally, and socially acceptable manner that meets the demands of an increasing population (1). In beef cattle, producing more protein via increased weight gain has relied traditionally on nutritional and genetic selection strategies. Through dietary modification and genetically enhanced breeding programs, producers have improved both weight gain and the efficiency of nutrient utilization in beef enterprises. However, the nutritional status of beef cattle and other ruminants is influenced by many factors, including diet, animal management, host genetics, and the diverse symbiotic microbiota colonizing the gastrointestinal tract. Appropriately, throughout the past decade, focus on microbial impacts on beef nutrition and feed efficiency has increased. Because the gut microbial communities of cattle are critical for the degradation of low-quality feedstuffs into glucogenic, lipogenic, and aminogenic precursors involved in energy metabolism in cattle and sustenance via other vital nutrients, further characterization of the complex matrix between the host and its ruminal and lower gastrointestinal microbiota holds promise for optimizing bovine production.

The gut microbiota of ruminants likely exceeds the compositional and functional diversity of their monogastric counterparts, converting the energy from forages to high-quality protein and considerably affecting production phenotypes, such as feed efficiency. Several of the first studies to examine the relationship between the gut microbiome and beef cattle feed efficiency were initially conducted as part of the USDA-NIFA National Program for Genetic Improvement of Feed Efficiency in Beef Cattle. This work demonstrated connections between microbial communities of the rumen, jejunum, cecum, and distal colon/rectum among steers and feed efficiency (2). These results were based on a feed efficiency metric taking into account the greatest deviation of average daily gain (ADG) and average daily feed intake (ADFI) within each Cartesian quadrant (3). In the rumen, significantly increased relative abundances of *Firmicutes* taxa were observed in the group of steers with greater ADG and less ADFI (i.e., the feed-efficient group), indicating that increased feed efficiency is correlated with more abundant *Firmicutes* populations. This association was intriguing because the *Firmicutes*-to-*Bacteroidetes* ratio is often implicated in human obesity research (4) and has been associated with fat deposition increases and energy harvesting in dairy cattle (5). Other taxa were also significantly associated with cattle feed efficiency in the rumen. Collectively, these microbes play important roles in the fermentative and cellulolytic capacity of the rumen based upon their putative functions. Throughout the gut, numerous genera and species were linked to feed efficiency in steers, as well as ADG, ADFI, and their interaction (2). Jejunal bacterial analyses revealed *Butyrivibrio* as the genus of greatest relative abundance that differed among the feed efficiency groups (6). Members of this genus were frequently identified as being associated with feed-efficient steers. These butyrate-producing, hemicellulolytic bacteria ferment a wide range of sugars and are important sources of energy for intestinal epithelial cells (7). In the cecum, many taxa of the family *Lachnospiraceae* were identified in feed-efficient steers, such as *Butyrivibrio*, *Pseudobutyrivibrio*, and *Blautia*, again signifying importance to the energy available to host intestinal tissues (2). Colonic bacteria, such as *Faecalibacterium*, were indicative of feed-efficient steers (2), and this has been associated with increases in weight gain and reduced incidences of diarrhea in calves (8). *Faecalibacterium* abundances, therefore, may be important for maintaining weight and reducing enteric infections in growing animals. These collective studies were significant to initially define the microbial impact throughout the rumen and lower gastrointestinal tract on beef cattle feed efficiency, while also highlighting the potential influence of individual microbial species on phenotypes, rather than global microbial changes.

Feed efficiency is also affected by common production practices. In cattle operations, producers routinely supplement diets with antimicrobials to improve animal health and feed efficiency. Rumen microbiology has been a valuable tool to determine the factors influencing variations in feed efficiency influenced by antimicrobials. In feedlot operations, the feeding of ionophores, such as monensin, is common and has been shown to improve feed efficiency and increase weight gain. As an added benefit, monensin is thought to reduce enteric methane production through its lethal interference with ion flux in Gram-positive bacteria, reducing the availability of substrates to rumen methanogenic archaea. The reduction in methane production is also considered to improve feed efficiency, because carbon can be used for animal growth rather than methane. With improved gas analyses, sequencing technologies, and sophisticated bioinformatics tools, current research has not supported this model of monensin action. Examination of the bacterial communities in bred heifers fed monensin in confinement showed that treatment with the ionophore impacted the abundances of individual bacterial taxa, such as populations of cellulolytic *Ruminococcus* taxa (9). Yet, the proportion of Gram-positive to Gram-negative bacteria did not differ with supplementation. Furthermore, methanogen abundances and methane and carbon dioxide emissions were not impacted by monensin supplementation (9). The use of heifers in this study was important because the typical 15-month life span of market steers reduces the potential for long-term methane mitigation. Heifers and cows remain in the herd longer and contribute to a greater proportion of the methane attributed to beef cattle. Although still important for improving feed efficiency in steers, the work demonstrated that ionophore use in heifers may be ineffective at reducing methane emissions long-term and that the mechanism of action of monensin is more complex than previously believed.

Ruminant microbial community analyses have surpassed the age of microbial taxon cataloguing, and researchers have begun to use multidisciplinary tools to determine mechanisms driving variations in feed efficiency. Microbial metabolism impacts the yield of energy from the feed. Thus, untargeted metabolomics can be implemented to profile host nutrient utilization, adding a link to the host-microbe network. Recent research has established that Angus steers varying in feed efficiency exhibit different serum metabolomic profiles and metabolites. Particularly, pantothenate is increased in feed-efficient steers, which is a key metabolite involved in intermediary metabolism (10). All living organisms require pantothenate, and it is essential for the production of coenzyme A (11). Coenzyme A is a carrier molecule important for the synthesis and oxidation of fatty acids, aiding metabolic functions in both anabolic and catabolic pathways. Because it facilitates numerous oxidation pathways—notably the tricarboxylic acid (TCA) cycle—coenzyme A is important in carbohydrate and fat metabolism, ultimately linking its abundance to weight gain, muscle growth, and animal performance. Pantothenate is acquired by ruminants via absorption directly from the feed, endogenous synthesis, or microbial production, and increases in its abundance provide an association with microbial metabolism. The identification of pantothenate in the serum of feed-efficient steers also suggests that this metabolite may be relevant to the development of biochemical markers for feed efficiency from common blood samples. Further investigations of pantothenate in cattle have identified microbial markers for both feed efficiency and pantothenate. In the same animals, ruminal proportions of the bacterial class *Flavobacteria* (*Bacteroidetes*) were enriched in feed-efficient steers (12). Greater proportions of pantothenate-producing bacteria, such as *Flavobacteria*, may result in improved nutrient utilization in steers.

**FIG 1:**
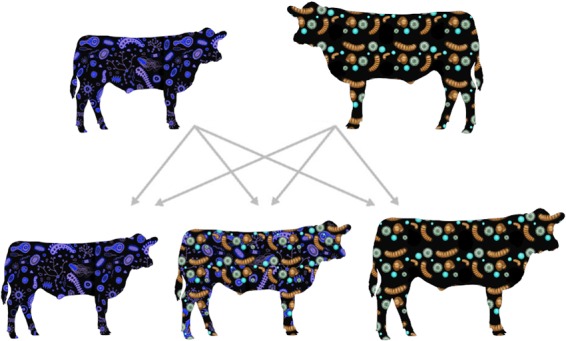
Rumen and gut microbiomes as heritable phenotypes. The genetic selection for heritable rumen and gut microbes permits opportunities to manipulate microbiomes long term for lasting cattle production outcomes, such as feed efficiency and body weight.

Ongoing host-microbe symbiosis research will hopefully better address whether the rumen microbiome drives changes in the host or the host drives changes to the rumen microbiome. Although these symbioses will likely be defined along a spectrum of gene-environment interactions, evidence from quantitative genomics, particularly genome-wide association studies (GWAS), demonstrates that host genetics is important in determining the composition of the gut microbiome of mice and humans (13). The field of rumen microbiology is currently undertaking similar investigations to define the bovine genome-microbiome relationship, with the ultimate goal of genetically manipulating the rumen microbiome for improvements in production efficiency (14). Historically, advances in cattle production efficiency have relied on genetic selection and improvement technologies. These host-centric efforts have slowly pushed the boundary on production outputs. Enhancing the genetic selection of production-relevant traits should also extend to microbiomes, as their collective function elicits discernible phenotypes. Advances in the field of rumen microbiology will undoubtedly rely on interdisciplinary systems-based concepts. Considering gut microbiomes as heritable phenotypes, researchers have already begun to identify heritable microbes and microbial features in human and animal systems (15). Once the influence of host genetic variation on rumen microbiome composition and heritable microbiota is established, we will be able to identify persistent microbial taxa and functions that are characteristic of efficient beef cattle (Fig. 1), thereby directly aiding in efforts to genetically select or permanently modify the ruminal microbiome. Within the next 5 years, researchers will further define the relationship between gut microbiomes and the genetics of their ruminant hosts, unraveling an intricate network that paves the way for the genetic selection of heritable microbes and keystone microbial species. These efforts will rapidly advance microbial ecology research and production agriculture to efficiently and sustainably produce high-quality protein for human consumption to achieve a food-secure global population.
